# 

**Published:** 1900-11

**Authors:** 


### Society Notes.

### Brazos Valley Medical Association.

The Brazos Valley Medical Association of Texas will hold its tenth semi-annual meeting, at Cameron, on the second Tuesday and Wednesday in November, the 13th and 14th days. A most excellent program is presented. A goodly number of physicians are looked for and a pleasant time expected.

W. B. BRIGGS, Secretary.

CAMERON, TEXAS, Oct. 30, 1900.

*Dear Doctor:*

The physicians and citizens of Cameron earnestly request the presence of yourself and ladies at the Tenth Semi-Annual Meeting of the Brazos Valley Medical Association, which convenes in our city on November 13th and 14th, 1900. Lay aside the cares of this busy season and mingle with your fellow laborers. It will do you good. The gates of our city are wide open and we welcome you.

Sincerely yours,

COMMITTEE OF ARRANGEMENTS.

PROGRAM.

TUESDAY.

1. Invocation, Rev. James Kilgore.
2. Address of Welcome on Behalf of City, Mayor J. M. Ralston.
3. Address of Welcome on Behalf of Physicians of Cameron, Dr. M. K. Lott.
4. Response by President of Association.
5. Section Work.
6. Musical Entertainment by Local Telant, at the Auditorium, 8 p. m.

WEDNESDAY.

A. M.

1. Section Work.

P. M.

2. Section Work.
3. From 4 to 6, a reception in honor of the visiting ladies, at the residence of Dr. and Mrs. E. S. Ferguson.
4. At 8:30 p. m., reception and banquet to our guests, at the Auditorium.

